# Health Risks Associated with Exposure to Filamentous Fungi

**DOI:** 10.3390/ijerph14070719

**Published:** 2017-07-04

**Authors:** Mary Augustina Egbuta, Mulunda Mwanza, Olubukola Oluranti Babalola

**Affiliations:** 1 Department of Biological Sciences, Faculty of Agriculture, Science and Technology, North-West University, Private Bag X2046, Mmabatho 2735, South Africa; olubukola.babalola@nwu.ac.za; 2 Southern Cross Plant Science, Southern Cross University, Lismore, NSW 2480, Australia; 3 Department of Animal Health, Faculty of Agriculture, Science and Technology, North-West University, Private Bag X2046, Mmabatho 2735, South Africa; mulunda.mwanza@nwu.ac.za

**Keywords:** mycotoxicity, infections, *Aspergillus*, *Fusarium*, *Penicillium*

## Abstract

Filamentous fungi occur widely in the environment, contaminating soil, air, food and other substrates. Due to their wide distribution, they have medical and economic implications. Regardless of their use as a source of antibiotics, vitamins and raw materials for various industrially important chemicals, most fungi and filamentous fungi produce metabolites associated with a range of health risks, both in humans and in animals. The association of filamentous fungi and their metabolites to different negative health conditions in humans and animals, has contributed to the importance of investigating different health risks induced by this family of heterotrophs. This review aims to discuss health risks associated with commonly occurring filamentous fungal species which belong to genera *Aspergillus*, *Penicillium* and *Fusarium*, as well as evaluating their pathogenicity and mycotoxic properties.

## 1. Introduction

Over the years, from the 1970s until the present, a range of filamentous fungi species belonging to different genera are being mentioned in relation to many infections affecting different organs such as the eyes, ears, nasal cavity, nails, skin, respiratory tracts, internal organs, etc. [1,2,3,4,5]. Filamentous fungi species occur commonly in the environment due to the ability of this group of fungi to grow on almost any substrate and under harsh conditions [6], and, they are able to produce spores at low temperatures which are distributed in the air. These fungi species are heterotrophic and saprophytic organisms deriving nourishment and energy from dead organic matter [7] and possessing the ability to synthesize a variety of natural products as primary and secondary metabolites. *Aspergillus*, *Fusarium*, *Penicillium*, *Cladosporium*, *Acremonium*, *Alternaria* and *Curvularia* are some of the genera of fungi which belong to the family of filamentous fungi [8] with the *Aspergillus* species reportedly most abundant and widely distributed globally [9].

Although some filamentous fungi, especially those belonging to genus *Aspergillus* such as *Aspergillus niger* and genus *Penicillium* such as *Penicillium citrinum* are being implemented in the food and pharmaceutical industries as a result of some metabolites they produce [10,11], these filamentous fungi have also been reported in association with infections and disease [12,13]. Some filamentous fungi have been reported to cause both superficial infections in the case of skin and nail infections, as well as invasive infections particularly in immuno compromised individuals [1,3,14,15,16,17].

Production by filamentous fungi of mycotoxins, secondary metabolites which have many negative health effects, is another contributory factor to the health risks posed by the fungal species belonging to this family. Mycotoxin production by filamentous fungi which usually occurs in response to certain conditions such as humidity, temperature etc. [18,19]; is the main factor for mycotoxicosis by filamentous fungi in humans and animals. These mycotoxins have negative impacts on the agricultural industry as well as being found in association with a variety of human and animal diseases such as oesophageal cancer, liver cancer and benign endemic nephropathy (BEN) in humans, as well as equine leuco-encephalo malacia (ELEM), hormonal disorders, immunosuppression and even deaths in animals [20,21,22,23]. 

Although, most of these filamentous fungi have been used and are still manipulated biotechnologically in the food industry and pharmaceutical/medical industry, it is of utmost importance to look extensively at the health risks of these filamentous fungi especially those that commonly occur in the environment, whether in food or in the atmosphere. In order to contribute to knowledge of health risks associated with filamentous fungi species, this review aims to discuss the ability of these fungi to exert negative health effects, especially in humans giving cognition to their mycotoxic, cytotoxic, DNA damaging and immune-suppressing and properties.

## 2. Toxin Production by Filamentous Fungi

As was mentioned earlier in the text, many species belonging to the filamentous fungi group produce the secondary metabolites known as mycotoxins which are substances that in most cases, have toxic effects when humans and animals are exposed to them [24]. These mycotoxins include aflatoxins, ochratoxins, fumonisins, trichothecenes, deoxyivalenol, zearalenone, gliotoxin, amongst others. Over 300 different mycotoxins are synthesized by filamentous fungi [25] and mycotoxin production is common in species of the genera *Aspergillus*, *Penicillium*, *Fusarium*, *Alternaria* and *Cladosporium* [26]. It is usually common to find one mycotoxin being synthesized by different fungal species and across genera, as is the case of ochratoxin A, which is produced by *A. niger*, *A. ochraceus* and *P. viridicatum*. There is also another situation where one fungal specie produces more than one mycotoxin, such as *F. verticilliodes* and *F. culmorum* which produce fumonisin B1, moniliformin, nivalenol, deoxynivalenol and other mycotoxins at the same time [27]. This is well illustrated in Table 1.

*Aspergillus* species are producers of a wide range of mycotoxins which includes the aflatoxins and sterigmatocystin produced by *A. flavus*; ochratoxin A, malformin, oxalic acid and fumonisin B2 produced by *A. niger*; viriditoxin and gliotoxin produced by *A. fumigatus* and patulin, tryptoquivalene and cytochalasin E produced by *A. clavatus* amongst others [24]. *Fusarium* species are known producers of mycotoxins such as fumonisins, acetoxyscirpenediol, moniliformin, nivalenol, enniatins, fusaric acid, and fusarin [26]. Other mycotoxins produced by *Penicillium* species include ochratoxin A, islanditoxin, penitrem, rubratoxin, rubroskyrin, rubrosulphin, rugulosin, citrinin, citreoviridin, gliotoxin, patulin, viopurpurin and viomellein [26]. 

According to Richard [21], mycotoxins synthesized by filamentous fungi have been conjecturally associated with diseases by modern day investigators and go back to times included in the writings of the Dead Sea Scrolls. These mycotoxins induce powerful biological effects of which a prolonged and continuous exposure either by ingestion or inhalation could result to harmful and negative health implications [29,30]. The aflatoxin group, which is one of the five most important occurring mycotoxins [31] comprise of aflatoxin B1, B2, G1 and G2. They are primarily hepatoxic toxins targeting mainly the liver with aflatoxin B1 (AFB1) being the most potent and classified as a human carcinogen (Group 1) by the international Agency for Research on Cancer (IARC) [32]. Ochratoxin A is another major mycotoxin that has been classified as a possible human carcinogen by the IARC. Targeting mainly the kidney, this toxin is nephrotoxic, teratogenic, carcinogenic and immuno-suppressive in many animal species [33]. Other major mycotoxins such as fumonisins, deoxynivalenol and zearalenone also induce carcinogenic, teratogenic, mutagenic, genotoxic and immune suppressing effects in humans and animals [21,25,34].

There have been severe negative health cases mentioned with regards to mycotoxin poisoning in most parts of the world in humans and animals. One of such cases is the Balkan Endemic Nephropathy, where it is reported that OTA was associated with this disorder in the Balkan areas of south-eastern Europe [21,23]. Another of such cases was acute aflatoxin exposures reported to be associated with epidemics of acute hepatitis in areas of China and Africa which resulted in deaths [25]. Some of the metabolites have been classified as carcinogens by the international Agency for Research on Cancer (IARC) because of the negative health effects (as indicated in Table 2) they exert on different organs of the body [31,32,35,36].

## 3. Infections/Diseases Induced by Filamentous Fungi

Filamentous fungal species are widespread in the environment and as such have been reported in association with some human and animal infections and diseases [5,65]. A host of fungal infections have been reported in association with some genera of the filamentous fungi such as *Aspergillus*, *Fusarium* and *Penicillium*. As mentioned earlier, these groups of fungi produce metabolites that have been reported in association with a range of diseases and infections (Table 3). These fungal genera induce infections in specific manners with preferences for specific organs and parts of the body [15].

### 3.1. Aspergillus Species

Within the genus *Aspergillus*, over 20 species have been reported as causative agents of opportunistic infections in man. Aside from the production of mycotoxins, *Aspergillus* species are well-known to play a role in three different clinical settings in man: opportunistic infections, allergic states and toxicoses [65] with the most route of transmission of spores being by inhalation [15]. *Aspergillus* species have been mentioned in association with a range of infections which include allergic bronchopulmonary aspergillosis (ABPA), aspergilloma, chronic necrotizing aspergillosis (CNA) and invasive pulmonary aspergillosis (IPA) generally referred to as aspergillosis which usually affects the lungs and could also spread to other parts of the body. Aspergillosis usually affects people with existing health conditions especially those with damaged lungs and suppressed immunity [76] and the use of drugs and medications have contributed to reducing immunity in even healthy individuals. Common signs associated with aspergillosis include difficulty in breathing, increased thirst, diarrhoea and fever [77]. Out of all the species belonging to the genus *Aspergillus*, *A. niger*, *A. flavus*, *A. fumigatus*, *A. versicolor* and *A. nidulans*, are some of the few *Aspergillus* species possessing the ability to induce disease and infection [78]. Although *A. ochraceus* has the ability to produce mycotoxins, there is limited report of this specie inducing any form of infection in man or animals.

#### 3.1.1. *Aspergillus fumigatus*

*Aspergillus fumigatus* is the most commonly occurring aerial pathogen with life threatening properties and commonly isolated from blood and tissues of individuals exhibiting symptoms associated with *A. fumigatus* infection [68,78]. *Aspergillus fumigatus* has been reported in association with a range of pulmonary infections as it has been reported to produce metabolites such as polypeptide allergens that are responsible for asthma and rhinitis [68]; also producing mycotoxins such as gliotoxins that have negative health effects on humans and animals as well as β-1,3 glucans that are known modulators of the immune system [79,80]. *Aspergillus fumigatus* has been reported as a source of increased immune-suppression and possibly mortality in immuno-compromised individuals, with the characteristic of immune-suppression attributed to the production of the toxic metabolite gliotoxin during hyphal growth under specific favourable conditions [80]. A recent study by [81] showed the production of proteins and other allergens by *A. fumigatus* which is also contributory to the ability of this specie of fungi to induce infection and disease.

#### 3.1.2. *Aspergillus flavus*

*Aspergillus flavus*, commonly isolated *Aspergillus* specie from soil and contaminated food commodities is also a common fungal pathogen associated with a range of fungal infections. A common causative agent in invasive aspergillosis and superficial infections, *A. flavus* has been associated with clinical syndromes such as chronic granulomatous sinusitis, keratitis, cutenous aspergillosis, wound infections and osteomyelitis [14,67]. This fungal specie has been reported to cause adverse health conditions in immuno-compromised individuals which could be sometimes fatal [15] and can be virulent in healthy individuals who are exposed to the spores. A case of severe penile *A. flavus* infection which had started as a rash and was consequently followed by selling, purulent secretions, skin erosion and bleeding on the glans was reported by Li et al. [82] from the Huashan Hospital (Fadan University, Shanghai, China). This was a case of very severe cutaneous aspergillosis which had to be treated using an antifungal agent and plastic surgery to close the wound caused by the infection. 

#### 3.1.3. *Aspergillus versicolor*

This slow growing *Aspergillus* specie is commonly found in damp indoor environment and on food products [83]. Apart from the production of hepatoxic and carcinogenic mycotoxin sterigmatocystin [84], this opportunistic pathogen has been reported to contain more than 20 allergens and irritating particularly the nose, eyes and throat [69]. *Aspergillus versicolor* has been reported to be another causative agent of aspergillosis with the fungi being a major cause of onychomycosis (a fungal infection of the nails) [85]. *Aspergillus versicolor* is also a causative agent of invasive aspergillosis and this was showed in a case study by [70]. An immuno-competent patient on mechanical ventilation support was diagnosed to have invasive pulmonary aspergillosis due to *A. versicolor* which eventually culminated in death of the patient due to treatment failure. 

#### 3.1.4. *Aspergillus candidus*

*Aspergillus candidus*, a common contaminant of grain dust and common producer of potent cytotoxic substances like *p*-terphenyl metabolites and terpenins [86,87] is reported to be a cause of respiratory disease in humans. This fungus has been identified as a potential respiratory hazard for individuals who are constantly exposed to it, even in immune competent individuals. A range of infections have been attributed to this *Aspergillus* specie which includes invasive aspergillosis, otomycosis, brain granuloma, onychomycosis, allergic alveolitis, and mycotoxicosis [1,66].

#### 3.1.5. *Aspergillus niger*

Possessing the ability to grow on a wide variety of substances, *A. niger* is a common contaminant of food, soil and indoor environment. Although its spores are widespread the fungus has been reported to a less likely cause of human disease in comparison to other *Aspergillus* species [12]. *Aspergillus niger* normally invades tissues which have already been rendered susceptible by bacterial infections, physical injury or accumulation of cerumen in the external auditory canal. Along with other *Aspergillus* species, *A. niger* is another causative agent of otomycosis, a superficial fungal infection in the ear, throat or nose that can be sub-acute or chronic. As is the case with most filamentous fungal species, immune compromised individuals are also susceptible to *A. niger* infections causing in most cases invasive pulmonary aspergillosis [3,12] characterized by chronic productive cough and coughing up blood.

### 3.2. Fusarium Species

This genus of filamentous fungi which contains over fifty species and is commonly found occurring in the soil and in association with plants such as cereals and grains is a causative agent of superficial and systemic infections. The fungi can infect humans and animals and infection can occur through inhalation of air-borne conidia and through cuts/breaks in the skin [74]. Along with causing a range of opportunistic infections, some *Fusarium* species produce mycotoxins which affect human and animal health if the enter the food chain. Infections caused by *Fusarium* species are generally referred to as fusariosis and the form of this condition fusariosis is largely dependent on the immune status of the host and the route of entry of the infection [88,89].

Among immunocompetent hosts, the common *Fusarium* infections are keratitis and onychomycosis with other less common conditions such as sinusitis, pneumonia, thrombophlebitis and fungemia [89]. With immunocompromised individuals, those at high risk of fusariosis are those with prolonged and profound neutropenia as well as T-cell immunodeficiency [90]. Within this genus, not all its species possess the ability to induce disease or infection with only a few causing infections such as *F. verticilliodes*, *F. solani complex*, *F. oxysporum*, *F. proliferatum* amongst others with *F. solani* being the most frequent [89].

#### 3.2.1. *Fusarium verticilliodes*

Commonly contaminating maize, rice, other cereals and grains, *F. verticillioides* is one of the producers of the mycotoxin fumonisins which have been reported to have adverse negative health effects including oesophageal cancer [41,91,92]. This human pathogen is more of a common causative agent of infection in immune-compromised individuals than in immune-competent individuals and this is because one of the important predisposing factors to *F. verticillioides* infection is severe prolonged neutropenia [73,74]. Infection by *F. verticilliodes* has been reported to be in immune-compromised individuals who have undergone a major organ re-transplant due to rejections by their bodies [73,90,93]. 

Another infection induced by *F. verticillioides* is *Fusarium* keratomycosis, a fungal infection of the cornea which is characterised by red and painful eyes with ulcers being present sometimes [72]. Symptoms associated with *F. verticillioides* infections include necrotic lesions on the skin mostly on the legs, fever and endothelial proliferation of internal organs [73,74,93,94].

#### 3.2.2. *Fusarium solani*

Most virulent of all *Fusarium* species and often reported in relation to fusariosis, *F. solani* is a *Fusarium* specie that commonly occurs in the environment [95,96]. *Fusarium solani* which also acts as a plant pathogen has been reported to induce a range of diseases and infections in both immune-competent and immune-compromised individuals. These diseases/infections include keratitis, endophtalmitis, onychomycosis, cutaneous infections, sub-cutaneous infections, arthritis, mycetoma, sinusitis and disseminated infections in immune-compromised individuals [16,71]. Although a variety of *Fusarium* species are causative agents of mycotic keratitis, *F. solani* is frequently reported in comparison to other species [65,95]. Cases of fungal infections such as mycotic keratitis due to *F. solani* have been reported as far back as the 1970s [4] with individuals developing corneal ulcers. Symptoms of *F. solani* infections include ulcers, fevers, skin lesions and organ membrane disruption.

#### 3.2.3. *Fusarium oxysporum*

*Fusarium oxysporum*, which comprises all the species, varieties and forms within a group called section *Elegans*, is a highly ubiquitous *Fusarium* specie possessing the ability to survive in different environments ranging from the desert, through the tropical and temperate forests to the grasslands [97]. Strains from this specie of fungi have been classified as non-pathogenic, human pathogenic and plant pathogenic [5,98]. The human pathogenic strains of *F. oxysporum* are known to cause infections in both human and animals whether immunocompetent or immunocompromised [16]. Infections such as keratitis, onychomycosis are some of the infections associated with *F. oxysporum* as well as localized necrotic diseases [16,99].

### 3.3. Penicillium Species

*Penicillium* species are diverse and widely distributed in the environment but despite their abundance and diversity in the environment, they are not commonly associated with human and animal infections [75]. With some of the few pathogenic species affecting plants, *P. citrinum*, *P. chrysogenum*, *P. digitatum*, *P. expansum* and *P. marneffei* are commonly associated with humans/animals and the mode of infection being mostly through inhalation and sometimes ingestion [13,100]. Diseases that are as a result of *Penicillium* infection by any *Penicillium* specie are generally referred to as penicilliosis. Species of this genus have been mentioned in association with infections such as keratitis, endophtalmitis, otomycosis, pneumonia, endocarditis and urinary tract infections [2].

#### 3.3.1. *Penicillium citrinum*

Being one of the commonly occurring *Penicillium* specie, this fungus is a plant pathogen as well as a human and animal pathogen. This fungus which also produces the mycotoxins ochratoxin and citrinin, has been reported in association with some fungal infections and other diseases which include keratitis, asthma and pneumonia [13,75] which in some cases could be fatal.

#### 3.3.2. *Penicillium marneffei*

This fungus is the third most common opportunistic pathogen with individuals suffering from AIDs in areas where it is endemic [75,100]. *Penicillium marneffei* is also a common fungus infecting individuals with haematological malignancies as well as individuals receiving immunosuppressive therapy when they are exposed to it. In areas like Southeast Asia, this fungus has continued to cause morbidity and mortality in HIV positive that either are unaware of their status or are not able to anti-retroviral therapy [13]. Individuals exposed to *P. marneffei* infections exhibit clinical manifestations such as weight loss, skin lesions, fungemia, pulmonary lesions, anaemia, cough and low-grade fever [13]. 

#### 3.3.3. Other Less Common Pathogenic Penicillium Species

*Penicillium digitatum*, *P. expansum* and *P. chrysogenum* are some of the other less common pathogenic fungi belonging to the genus *Penicillium*. Although they are not frequently mentioned in relation to penicilliosis, these fungal species cause infections in human that could be fatal. *Penicillium digitatum* which is a post-harvest plant pathogen was reported in association with fatal pneumonia [17]. *Penicillium chrysogenum* and *P. expansum* have been reported to be causative agents of necrotizing esophagitis, endophthalmitis, keratitis and asthma [13].

## 4. Cytotoxicity Induction by Filamentous Fungi

Cytotoxicity is the ability of a compound or substance to induce toxicity on cells. Many compounds are known to be cytotoxic to both human and animal cells thereby inducing reduction in cell viabilities, apoptosis or complete cell death (necrosis). Fungi and their metabolites are not left out in the group of cytotoxic compounds, especially to humans and animals. Cytotoxic actions of fungi isolates are reported to be either beneficial or a health risk. The beneficial cytotoxic effects of fungi isolates can be considered when these fungi act as endophytes, inhibiting the pathogenic effects other fungi species or bacteria in plants or food crops [101]. Another beneficial cytotoxic effect of fungi is the ability of some of these species to act as anti-tumour and anti-cancer agents thereby inhibiting the uncontrolled proliferation of cancer cells [102,103,104]. Some species belonging to the genus *Fusarium* have been reported to induce cytotoxicity in various cancer cell lines including HCT-116 cells (human colon carcinoma), MCF-7 cells (breast cancer cell), PC-3 cells (prostate cancer cell), A-549 cells (human alveolar basal epithelial cells), HeLa cells and HepG2 cells [105,106]. 

The negative cytotoxic effects of fungi species have been recorded in some studies involving normal human and animal cell lines. Species belonging to the *Aspergillus* genus such as *A. fumigatus*, *A. niger*, *A. flavus* and *A. ochraceus* induce reduced cell viability and cell death in cell lines exposed to them [107,108]. Aflatoxins, ochratoxins, gliotoxin and other mycotoxins which are produced by these *Aspergillus* fungi induce reduced cell viabilities in both human and animal cells at varying concentrations [25,109,110,111]. *Fusarium* species and their metabolites also induce cytotoxicity on both human and animal cell lines. Studies by Abeywickrama and Bean [112], Hameed et al. [113] and Langseth et al. [114] demonstrated the cytotoxic effects of *F. culmorum*, *F. acuminatum*, *F. graminearum*, *F. solani*, *F. equiseti*, *F. poae*, *F. sporotrichioides* and *F. tricinctum* on different mammalian cell lines inhibiting cell viability and even causing cell death. The cytotoxic effects of *Fusarium* species could be attributed to some secondary metabolites they produce during the course of their growth in response to stimuli. Mycotoxins such as FB1, zearalenone, enniatins, T-2 toxin and the trichothecenes produced by *Fusarium* species exert cytotoxic effects on human and animal cells at varying concentrations and duration of exposure [57,115,116,117,118].

Cytotoxic effects by *Penicillium* species are not as common as the other genera of fungi mentioned although a few *Penicillium* species have shown in past studies that they have the tendency to inhibit cell proliferation and cell viability. In their study, Shah et al. [119] reported the cytotoxic effects of *P. verrucosum* on normal and cancer cells, resulting in reduced cell viabilities of normal cells and inhibiting cell proliferation in cancer cells. A study by Geiger et al. [120] showed that *Penicillium* species isolated from shell-fish were able to exert cytotoxic effects on cell lines exposed to them posing a health concern for the consumers of the product. *Penicillium* species also produce mycotoxins ochratoxin A, citrinin, patulin and penicillinic acid, which have cytotoxic effects on mammalian cell lines resulting in reduced cell viability depending on concentration and duration of exposure [111,121,122].

## 5. Immunosuppression by Filamentous Fungi

Immunosuppression which is an act that brings about a reduction of the efficacy of the immune system occurs in two ways; deliberate immunosuppression where the activity of the immune system is reduced by use of immunosuppressive drugs or immunosuppressant and in deliberate immunosuppression whereby the efficacy of the immune system is reduced as a result of factors such as aging, disease, malnutrition and infection [123]. As a result of the different health effects of these filamentous fungi species and the metabolites they produce, this group of micro-organisms contribute to reduce the efficacy of the immune system of mammals upon continuous exposure to them. 

The after effects of cytotoxicity of fungi species to cells and its components can be contributory their immunosuppressive activity as was reported by Kamei et al. [108] of the cytotoxicity of filamentous fungi species to macrophages which help initiate defence mechanisms in cells. Corrier [124], also reported that metabolites produced by filamentous fungi induced a reduction in immune activity of cells by suppressing T and B lymphocyte activity, immunoglobulin production, anti-body production, complement/interferon activity and impairing macrophage effector cell function. Also, a study by Pahl et al. [125] showed that gliotoxin produced by *A. fumigatus* could promote immunosuppression by inhibiting/interfering with the activation of transcription factors that are involved in T cell activation. A recent study by Fontaine et al. [126] has also contributed to substantiate the immuno-suppressive ability of some fungi species. It was discovered that *A. fumigatus* secretes a polysaccharide Galactosaminogalactan (GG) which favours aspergillosis by inducing neutrophil apoptosis. These neutrophils which act as pathogen destroyers are killed by the secretion of this polysaccharide and thereby resulting in immunosuppression in the host.

## 6. DNA Damage by Filamentous Fungi Species

The molecule which encodes all the genetic information necessary for proper development and functioning of a living organism known as the DNA is a very vital part of any living organism. The DNA can be damaged as a result of alterations in the chemical structure of the DNA in the form of a break in the DNA strand, a chemically changed base or a missing base in the DNA backbone. Alterations in the DNA of a living organism can result in consequences such as mutations in the cell and genomic instability (altered gene functions and expressions) which on the long run could contribute to cancer progression in cells [127]. Filamentous fungi are one of the factors responsible for DNA damage alongside chemical carcinogens, metabolites, ultraviolet radiation and polycyclic aromatic carbons. Producton of metabolites such as mycotoxins places filamentous fungi in the group of possible causes of DNA damage in living organisms. Many experimental studies have proved the ability of mycotoxins produced by filamentous fungi *Aspergillus*, *Fusarium* and *Penicillium* to induce DNA damage both in human and animal cells [122,128,129,130]. 

One major reason DNA is susceptible to damage by mycotoxins is because the nucleophilic hetero-atoms in the organic-bases of nucleic acids (such as nitrogen and oxygen atoms) are susceptible to attacks by mycotoxins forming covalent bonds with them [129]. This association between the DNA and the mycotoxins results to the formation of DNA adducts which impair DNA synthesis and eventually increase the activation of oncogenes (genes involved at the beginning of cancer formation) [129,130]. Aflatoxin B1, ochratoxin A, sterigmatocystin and zearalenone amongst other mycotoxins which are produced by species of *Aspergillus*, *Fusarium* and *Penicillium* genera have been reported to bind to DNA in the cells to form DNA adducts thereby resulting in the activation of oncogene formation, inhibition of DNA synthesis, disruption of normal DNA replication and DNA polyploidy in cells [131,132]. A less commonly occurring specie, *Alternaria alternata* which belongs to the genus *Alternaria*, another member of the filamentous fungi family has been reported in association with human DNA damage. Producing the mycotoxin alternariol which interferes with the human DNA topoisomerase (enzymes that regulate excessive winding or insufficient winding of DNA) to result in DNA instability and subsequently DNA double strand break [128].

## 7. Presumed Synergistic Effects of Fungi

As mentioned earlier in the review, filamentous fungi commonly occur in the environment and it is always possible to find more than one species occurring in a particular place at a time. The possibility of synergistic health effects has come into view considering that these fungi species mentioned exert acute and chronic health effects individually. This assumption is because of several studies which have reported the synergistic effects of metabolites of filamentous fungi on human and animal cell lines. Studies by Mwanza et al. [121], Stoev et al. [111] and Creppy et al. [37] showed that a combination of FB_1_ produced by *F. verticillioides* and OTA produced mainly by *A. ochraceus* and *P. viridicatum* induced a greater decrease in cell viability of PHA (phytohaemagglutinin-p) stimulated human and swine blood lymphocytes when compared to the individual effects of the metabolites. A mixture of AFB1 and DON as well as AFB1 and ZEA resulted in synergistic cytotoxic effects on porcine kidney cells on a dose-dependent ratio [110]. Other studies by Wan et al. [118] and Dong et al. [132] indicated synergistic cytotoxic effects of *Fusarium* toxin (DON, ZEA, FB1, NIV, T-2 toxin and BEA) mixtures on normal swine jejunal epithelial cells and hamster ovarian cells resulting in loss of cell viability.

## 8. Future Prospects

It is imperative that research into infections by filamentous fungi species be geared towards understanding the molecular nature of the micro-organism when causing infections. This is because most fungal infections are treated by antibiotics and sometimes, in the long run, resistance to the drugs develop and may lead to death of the host [70]. There is limited knowledge about the molecular pathway of infection by most pathogenic fungi, even though much is known about the metabolites they produce. An in-depth knowledge of the molecular pathway of infection including genes expressed and enzymes activated by these fungi could contribute to drug design and manufacture. Also, due to the knowledge that more than one species occurring at a particular environment at a time, this could lead to co-infections by the different species and cause more adverse effects or less since they are already pathogenic individually. Knowledge of the genes expressed due to interaction of these fungi species, enzymes expressed could contribute to combating the scourge of fungal infections. It is therefore advised that more research into co-infections by filamentous fungi species be done, investigaing molecular nature of the fungi at time of infection in order to determine which genes or enzymes to target when designing drugs to combat fungal infections.

## 9. Conclusions

Considering the wide occurrence of a variety of filamentous fungi species in the environment notwithstanding their positive uses, the negative health effects of these filamentous fungi is something to be addressed with much interest. The assumption that infections caused by this group of micro-organisms seldom occur must not to be encouraged because whether we agree or not, these filamentous fungi cause an alarming number of detrimental infections that should not be overlooked.

## Figures and Tables

**Table 1:** Filamentous fungi species and mycotoxins produced.

Fungal Genera	Mycotoxins Produced
*Aspergillus*	
*A. carneus*	Citrinin
*A. clavatus*	Cytochlasin E, Patulin, Tryptoquivalene
*A. flavus*	Aflatoxins, Sterigmatocystin
*A. fumigatus*	Fumagilin, Gliotoxin, Verruculogen, viriditoxin
*A. nidulans*	Sterigmatocystin
*A. niger*	Malformin, Oxalic acid, Ochratoxin A
*A. ochraceus*	Ochratoxin A, Penicillinic acid, Destruxin
*A. terreus*	Citrinin, Citreoviridin
*A. ustus*	Austdiol, Austamide, Austocystin
*A. versicolor*	Cyclopiazonic acid, Sterigmatocystin
*A. parasiticus*	Aflatoxins
*Fusarium*	
*F. avenaceum*	Enniatins, Fructagenin +1, HT-2 toxin, Ipomeanine, Lateritin +1, Lycomerasmin +1, Moniliformin, Monoacetoxyscirpenol, Neosolaniol, Nivalenol, Sambucynin
*F. culmorum*	Deoxynivalenol, Fructagenin +1, HT-2 toxin, Ipomeanine, Lateritin +1, Lycomerasmin +1, Moniliformin, Neosolaniol
*F. equiseti*	Moniliformin, Nivelenol, Monoacetoxyscirpenol, Acetoxyscirpenediol, Acetyldeoxynivalenol, Acetylneosolaniol, Acetyl T-2 toxin, Avenacein +1, Beauvericin +2, Butenolide, Calonectrin, Deacetylcalonectrin, T-1 toxin, zearalenol, T-1 toxin, T-2 toxin
*F. nivale*	Deoxynivalenol diacetate, HT-2 toxin, Ipomeanine, Lateritin +1, Lycomerasmin +1, Moniliformin, Monoacetoxyscirpenol, Sambucynin
*F. oxysporum*	Moniliformin, Monoacetoxyscirpenol, Neosolaniol, Nivalenol, Acetoxyscirpenediol, Acetyldeoxynivalenol, Acetylneosolaniol, Acetyl T-2 toxin, Avenacein +1, Beauvericin +2, Butenolide, Calonectrin, Deacetylcalonectrin, zearalenone
*F. roseum*	Fructagenin +1, Moniliformin, Monoacetoxyscirpenol, Neosolaniol, NT-1 toxin, N-2 toxin
*F. solani*	Enniatins, T-1 toxin, T-2 toxin, Sambucynin, Scirpentriol
*F. verticillioides*	Fumonisins, Monoacetoxyscirpenol, Neosolaniol, Ipomeanine, Avenacein +1, Beauvericin +2, Fusaric acid, Fusarin
*F. graminearum*	Zearalenone, Yavanicin+1
*Penicillium*	
*P. viridicatum*	Ochratoxin A, Rubrosulphin, Viopurpurin, Viomellein
*P. citrinin*	Citrinin
*P. verrucossum*	Citrinin
*P. hirsutum*	Citrinin
*P. citreoviride*	Citreoviridin
*P. islandicum*	Islanditoxin
*P. expansum*	Patulin
*P.roqueforti*	Patulin
*P. griseofulvum*	Patulin
*P. claviforme*	Patulin
*P. crustosum*	Penitrem, Viomellein
*P. rubrum*	Rubratoxin
*P. brunneum*	Rugulosin
*P. kloeckeri*	Rugulosin
*P. rugulosum*	Sterigmatocystin, Rugulosin
*P. aurantiogriseum*	Viomellein

Source: [26,27,28].

**Table 2:** Common mycotoxins, their health effects and target organs.

Mycotoxins	Health Effects	Target Organs	References
Aflatoxins	Hepatotoxic and immune-suppressive	Liver	[29]
Ochratoxin A	Carcinogenic, teratogenic, Immuno-suppressive, nephrotoxic and causing upper urinary tract disease	Kidney, liver	[37,38,39,40]
Fumonisins	Carcinogenic, hepatotoxic, nephrotoxic, immunosuppressive	Gastro-intestinal tract (GIT), liver, kidney	[41,42,43]
Deoxynivalenol	Nausea, vomiting, diarrhea, reproductive effects and toxicosis	Reproductive organs, GI	[18,21,30]
T-2 toxin	Hepatotoxic, genotoxic and immune-suppressive	GIT, Immune system	[44,45]
Zearalenone	Carcinogenic, hormonal imbalance and reproductive effects	Reproductive organs	[19,46]
Nivalenol	Anorexic, immunotoxic, haematotoxic and genotoxic	GIT, immune system	[47,48]
Sterigmatocystin	Genotoxic, cytotoxic, immunotoxic and carcinogenic	Liver, immune system, kidney	[49,50]
Cyclopiazonic acid	Immunotoxic and hepatotoxic	Muscle, hepatic tissue and spleen	[51,52,53]
Moniliformin	Cardiotoxic, muscular disorders, immunotoxic	Heart, Kidney, and muscles	[54,55]
Enniatins	Immunotoxic, cytotoxic	Immune system	[56,57,58]
Gliotoxin	Immunotoxic, nephrotoxic, hepatotoxic and genotoxic	Kidney, liver, immune system	[59,60,61,62]
Citreoviridin	Teratogenic and immunotoxic	Not specific	[63,64]
Citrinin	Nephrotoxic	Kidney	[24]

**Table 3:** Infections induced by fungi species and organs they target.

Fungi Species	Target Organs	Diseases Induced	References
*Aspergillus candidus*	Respiratory tract, brain, ear and nails	Respiratory disease, otomycosis, onychomycosis, brain granuloma	[1,66]
*Aspergillus flavus*	Nails, respiratory tract, bone and eye	Sinusitis, keratitis, aspergillosis, osteomyelitis	[14,67]
*Aspergillus fumigatus*	Respiratory tract	Pulomonary infections	[68]
*Aspergillus niger*	Ears, throat and respiratory tract	Otomycosis, pulmonary aspergillosis	[3]
*Aspergillus versicolor*	Nose, eyes, throat, nails	Invasive aspergillosis, onychomycosis	[69,70]
*Fusarium oxysporum*	Eyes and Nails	Keratitis, onychomycosis	[16]
*Fusarium solani*	Eyes, respiratory tract, nails, skin and bone	Keratitis, sinusitis, endophtalmitis, onychomycosis, cutenous infections, mycetoma and arthritis	[16,71]
*Fusarium verticillioides*	Eyes, skin, internal organs such as lungs, etc.	Keratomycosis, skin lesions, proliferation of internal organs	[72,73,74]
*Penicillium citrinum*	Eyes and respiratory tract	Keratitis, asthma, pneumonia	[13,75]
*Penicillium marneffei*	Blood, skin and respiratory tract	Fungemia, skin lesions, anaemia	[13]
